# Quality of Basic Cardiopulmonary Resuscitation of Adults at Medium and High Altitudes, with and Without Conditioning: Study Protocol

**DOI:** 10.3390/jfmk11030253

**Published:** 2026-06-27

**Authors:** Joseba Rabanales-Sotos, Sonia Piñero-Sáez, Ángel López-González, Francisco García-Alcaraz, Jesús López-Torres-Hidalgo, Carmen María Guerrero-Agenjo, Jaime López-Tendero, Vicente Ferrer-López

**Affiliations:** 1 Nursing College, Universidad de Castilla-La Mancha (UCLM), 02006 Albacete, Spain; joseba.rabanales@uclm.es (J.R.-S.); sonia.pinero@uclm.es (S.P.-S.); francisco.galcaraz@uclm.es (F.G.-A.); vicente.ferrer@uclm.es (V.F.-L.); 2 Group of Preventive Activities, University Health Sciences Setting, Universidad de Castilla-La Mancha (UCLM), 02071 Albacete, Spain; jesusl@sescam.org (J.L.-T.-H.); cmguerrero@sescam.jccm.es (C.M.G.-A.); jaime.lopez@uclm.es (J.L.-T.); 3 Albacete Medicine School, Universidad de Castilla-La Mancha (UCLM), 02008 Albacete, Spain; 4 Castilla-La Mancha Health Service (SESCAM), Universidad de Castilla-La Mancha (UCLM), 02071 Albacete, Spain

**Keywords:** basic cardiac life support, heart massage, spectroscopy, near-infrared, high altitude, hypoxia

## Abstract

**Background**: Performing and maintaining high-altitude cardiopulmonary resuscitation (CPR) could pose a significant physical challenge for rescuers. The objective of this study is to analyse the effects of reducing the oxygen fraction at altitudes of 3000 m and 5000 m above sea level (asl), with and without conditioning to hypoxia, on the quality of resuscitation performed in adults. **Methods**: An analytical before–after study in which 56 students with a Degree in Nursing between 18 and 30 years old perform 10 min of resuscitation on a mannequin at different altitudes (670, 3000 and 5000 m asl) will be carried out. Subsequently completing an intermittent hypoxia conditioning programme, the participants will perform the resuscitation manoeuvres at previously referenced altitudes. Sociodemographics, CPR quality, self-perception CPR, adequate anthropometric data, physical condition, blood tests, oxygenation in muscular tissue, biceps, brachii and erector spinae, subjective perception of effort, anxiety levels and quality of resuscitation will be measured in all participants at different altitudes. **Discussion:** Although CPR is a submaximal effort manoeuvre, it is subject to being performed by anyone without motor disabilities. Our study will also provide evidence as to whether this characteristic continues to hold true in a hostile environment such as medium and high altitudes. Our study aims to demonstrate that the improvement in physical performance and recovery capacity induced by intermittent hypoxia conditioning programmes increases the quality of CPR in prolonged cardiac arrests and in adverse conditions, such as at high altitudes. The proposed study will contribute as a novelty to the estimation of the influence of high altitudes and conditioning on performing basic CPR manoeuvres. If the hypothesis turns out to be true, recommendations about the practice of moderate-intensity physical exercise could be incorporated into the CPR guidelines as one of the important aspects in the training of rescuers to conduct CPR.

## 1. Introduction

Cardiac arrest (CA) is understood to be the sudden, unexpected and potentially reversible interruption of spontaneous breathing and circulation, which can be caused by many different factors [1].

The CA time and the duration of cardiopulmonary resuscitation (CPR) manoeuvres are the major determinants of cerebral blood flow generated by chest compressions (CCs) [2,3].

With differences between and within countries, the annual incidence of out-of-hospital cardiac arrest (OHCA) in Europe ranges from 67 to 170 cases per 100,000 inhabitants [4]. In Spain, in more than half of the cases (56.7%), basic life support was performed before the arrival of Prehospital Medical Emergency Services [5]. Survival rates after OHCA range from 5.0% to 30.0% across different European countries, increasing considerably in those cases where early CPR is performed [6,7].

CCs are the recommended technique for enabling emergency artificial blood circulation. In order to perform CCs effectively and minimise the risk of injury, it is necessary to press the centre of the thorax, on the sternum, with sufficient force to produce a good artificial carotid or femoral pulse. The CCs must be regular, uniform and uninterrupted [1].

Once the first recommendations were developed, multiple investigations were conducted, culminating in the proposals made by the American Heart Association (AHA) and by the European Resuscitation Council (ERC) in 2025 [8,9]. To achieve blood flow through the CCs, the compression/decompression ratio must be 50:50, which is achieved by performing compressions at a rate of 100–120 per minute. The compression depth for adults is between 5 and 6 cm [9]. Ventilations should have a volume and air pressure sufficient to make the victim’s chest rise. Each ventilation should have a duration of approximately 1 s and an insufflated air volume of approximately 500–600 mL (6–7 mL/kg^−1^) [9].

There are several causes that affect survival rates after a CA. One of the main causes is the interruption of the CCs, which produces a rapid decrease in cerebral perfusion and the ineffectiveness of the efforts made [10].

All recommendations are based on the fact that anyone should start CPR in the case of an CA, and rescuers are told that they should only stop CPR when their exhaustion prevents them from continuing, even if the victim is not recovering [11,12,13]. Several authors report the ineffectiveness of the CCs performed when fatigue appears. However, the recommendations do not determine when resuscitation should stop, suggesting that fatigue appears between the first and fifth minutes of CPR, decreasing its quality [13,14,15,16,17]. Under optimal environmental conditions, the exercise performed by the rescuer during CPR is fundamentally aerobic and submaximal, being well tolerated from the cardiorespiratory perspective. The quality and duration of CPR depend on the fitness level of the rescuer [17]. However, the influence that other factors have on the correct performance of CPR has not been sufficiently established [18,19].

Liu S et al. [19] and López-González, A et al. [16,20] have reported that subjects with a higher BMI performed a greater number of compressions with adequate depth and compression/decompression ratio, as well as a mean percentage of correct CCs. However, they did not perform better on the other criteria of adequate CPR (compression rate, location, adequate release pressure on the chest). There are no studies that have shown the influence of age on the performance of CCs. However, as the physical capacity of an individual decreases with age, as quantified by the maximum oxygen uptake (VO_2_max), so does the ability to perform quality CPR [17,20].

The influence of extreme temperatures on the quality of the CPR has been studied by Barcala-Furelos et al. and Martín-Conty et al. [21,22], who concluded that working in these highly unstable environments implies an extra expenditure of energy on the part of the rescuers. In addition to performing CPR according to quality criteria, rescuers must do so in environments and conditions that directly affect their physiological and psychological capacity [23,24]. Previous studies on CPR using medium- and high-fidelity mannequins, demonstrated that, in both men and women, the trait anxiety score (STAI-TA) could be categorised as low, which indicates a low tendency to perceive situations as threatening. In contrast, state anxiety (STAI-SA) was high, reflecting a temporary emotional state of tension at the end of the CPR manoeuvres [24,25].

There are numerous high-altitude regions around the world. Asia is home to the world’s highest point, Mount Everest in the Himalayas, at 8848 m above sea level (m asl). Around 400 million people worldwide live at altitudes above 1500 m asl [26,27], while around 140 million live permanently at altitudes between 2500 and 5500 m asl [28,29]. In addition, it is estimated that more than 100 million tourists visit mountainous areas around the world each year [30,31]. People’s interest in outdoor activities at high altitudes, such as hiking, mountaineering, skiing, and even following of these sports by amateurs, has increased considerably in recent years [32].

Being and moving at high altitudes produces various physiological effects, including an increase in cardiac and respiratory workload, especially during exercise [33,34]. Performing and maintaining quality CPR at high altitude could pose a significant physical challenge for rescuers. Preliminary studies have shown that, in resuscitators, after five minutes of CPR at altitude, arterial blood oxygen saturation (SaO_2_) decreases, and heart rate (HR) and the rating of perceived exertion (RPE) increase. This fact negatively affects the number of effective CCs, ultimately decreasing the quality of the CPR [27,28,29,30]. These variations have been studied by various authors at altitudes between 3100 and 3700 m asl [34,35,36,37,38,39].

Currently, it is not uncommon to find sports teams and supporters that exceed these altitudes during practice or monitoring. However, there are no studies analysing the quality of CPR performed at very high altitudes or how CPR results change after programmed conditioning at that altitude.

Normobaric intermittent hypoxia (NIH) involves breathing air with a low oxygen concentration at sea level. Its goal is to produce improvements typically associated with altitude acclimatisation, such as enhanced performance (increased oxygen transport, greater muscle efficiency and improved metabolic function), as well as haematological benefits (increased secretion of erythropoietin, haemoglobin and erythrocytes) [40]. However, haematological changes should be interpreted with caution, as it is likely that most protocols do not achieve the level of hypoxia required to induce meaningful erythropoiesis or lasting increases in haemoglobin mass [41].

The main aim of the study is to analyse the effect of reducing the fraction of inspired oxygen (FiO_2_) at medium and high altitudes (3000 and 5000 m asl) on the quality of the basic CPR performed on adults. The secondary objectives are (a) to assess the perception of effort and levels of anxiety involved in performing CPR at these altitudes; (b) to determine whether there is an association between altitude, body mass index (BMI) and physical fitness and the proportion of correct CCs achieved at the proposed altitudes; (c) to analyse the muscle oxygen saturation (SmO_2_) values during CPR at the proposed altitudes; (d) to determine the blood variations produced (lactate, haematocrit (Hct), haemoglobin (Hgb), creatine phosphokinase (CPK), and myoglobin (Mb)); and (e) to assess the quality of the CPR components (CCs and ventilations), the ratings of perceived exertion (RPE), and associations between BMI, SmO_2_ and anxiety levels at 670, 3000 and 5000 m asl after an adaptation programme to intermittent hypoxia.

## 2. Materials and Methods

**Figure 1:**
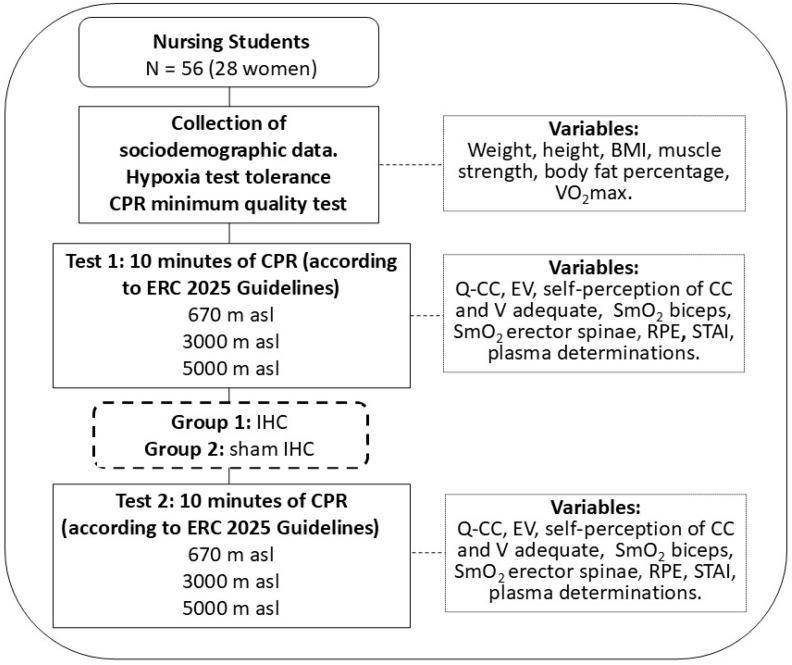
Flow chart. m asl: m above sea level; IHC: intermittent hypoxia conditioning; BMI: body mass index; VO_2_max: peak or maximum oxygen uptake; Q-CC: quality CC; EV: effective ventilation; SmO_2_: muscle oxygen saturation; RPE; rating of perceived exertion; STAI: State–Trait Anxiety Inventory.

A randomised, controlled, parallel-group experimental study will be conducted at the Faculty of Nursing of Albacete, University of Castilla-La Mancha, Spain (Figure 1). After, the participants will perform CPR manoeuvres on a mannequin at different altitudes (670, 3000 and 5000 m asl). Participants will be randomly assigned to one of two groups: (1) an intermittent hypoxia conditioning group (IHC), and (2) a sham conditioning control group. Subsequently, the participants will perform the CPR manoeuvres again at the aforementioned altitudes.

The population object of the study will be students with in their second year of the Degree in Nursing from the Faculty of Nursing of Albacete, University of Castilla-La Mancha, aged between 18 and 30 years, who agree to participate voluntarily after they understand the objectives of the study.

### 2.1. Inclusion and Exclusion Criteria

Inclusion criteria will include the ability to read and write in Spanish to complete the sociodemographic questionnaire and the ability to perform the ergospirometry test. The exclusion criteria will exclude those performing physical activity with an intensity >9 METS/min more than three times a week and those for whom the anamnesis advises against sports, as well as evidence of intolerance to altitude (determined with physiological parameters during the simulation of CPR at height). In addition, the following conditions were considered contraindications for inclusion in the study: chronic or acute bacterial infections, severe psychiatric disorders, neoplastic diseases, unstable or uncontrolled hypertension, severe balance problems, altitude sickness, unstable cardiovascular disease, chronic obstructive pulmonary disease, pregnancy, and other mental disorders or alterations that would prevent participants from taking the tests.

### 2.2. Ethical Considerations

All participants will agree to participate with written informed consent. This study respects the ethical principles of the Declaration of Helsinki. The protocol for the study was approved by the Ethics Committee for Investigation with medicinal products of the Integrated Care Management of Albacete, Spain (5 October 2021, code 2021-094).

### 2.3. Sample Size

Participants will be recruited using a convenience sampling approach among eligible nursing students. After baseline assessment, participants will be randomly allocated to one of two parallel groups: (1) an intermittent hypoxia conditioning group or (2) a sham control group. The random allocation sequence will be generated using a computer-based random number generator with a 1:1 allocation ratio. To ensure balance between groups, block randomisation will be applied with variable block sizes. Allocation concealment will be guaranteed using sealed, opaque, and sequentially numbered envelopes prepared by an independent researcher not involved in participant recruitment or assessment.

The assignment of participants to study groups will be conducted by a researcher not involved in outcome assessment or data analysis. Due to the nature of the intervention, participants and intervention supervisors cannot be blinded to group allocation. However, outcome assessors and data analysts will be blinded to group allocation to minimise detection and analysis bias. Specifically, outcome assessors will evaluate CPR performance using anonymised, coded data with no group identifiers, ensuring that group allocation remains concealed. The randomisation sequence generation and allocation concealment will be performed by an independent researcher, who will have no involvement in outcome assessment or data analysis.

To control for potential confounding variables, a multivariate model (multiple linear regression) will be applied. The change in Q-CC following the intervention will be considered as the dependent variable, while potential confounders (e.g., age, sex, BMI, physical fitness, and blood parameters) will be included as covariates. Interactions between these variables and altitude conditions will also be explored.

The sample size calculation is based on an expected baseline proportion of correct compressions of 52%, according to a previous study [16]. Using Epidat 4.2 software, assuming paired comparisons, a 95% confidence level, and 80% statistical power, a total of 56 participants is required to detect an increase in the proportion of correct compressions to 78%. To account for potential dropouts or non-responses, the total sample size will be increased by 10%.

Given the need to assess specific physiological parameters, including ergospirometry and simulated high-altitude exposure, participants will be recruited based on feasibility. A balanced distribution by sex (approximately 50% per group) will be ensured.

### 2.4. Interventions

#### Training CPR Programme

Participants without prior CPR training will undergo a standardised training programme designed in accordance with current European Resuscitation Council (ERC) guidelines. The training programme will consist of: (1) a theoretical session (60 min), including basic life support principles, recognition of cardiac arrest, and CPR sequence; (2) a practical hands-on training session (120 min), during which participants will practice chest compressions and ventilations using feedback-enabled mannequins under instructor supervision; and (3) a supervised skill consolidation session (30 min) focused on correcting technique and ensuring adherence to guideline criteria (compression depth, rate, recoil, and ventilation quality). The total training duration will therefore be approximately 3.5 h. Training will be delivered by certified instructors following a structured protocol to ensure standardisation across participants. Objective feedback devices will be used to facilitate motor skill acquisition and improve CPR quality performance. This training duration is consistent with previous literature demonstrating that structured sessions of at least 2–4 h are required for adequate acquisition of basic CPR skills and retention of performance quality.

Stage 1: An initial baseline assessment will be carried out in all participants (*n* = 56), consisting of collection of parentage data and anamnesis, as well as the measurement of physiological variables at rest (resting electrocardiogram and pulmonary function test), which will allow ruling out the existence of pathologies that contraindicate the performance of ergospirometry. Other measurements include diastolic blood pressure (DBP) and systolic blood pressure (SPB), anthropometry, ergospirometry to determine the maximal HR achieved and VO_2_max, muscle strength, and initial hypoxia tolerance test.

Stage 2: All participants (*n* = 56) will perform 10 uninterrupted minutes of CPR (with a CC/ventilation ratio of 30:2) on a mannequin with a quality measurement device for CTE and ventilations (Q-CPR) in the following situations:

Test M1: CPR at 670 m asl, FiO_2_ of 0.21.

Test M2: CPR at normal pressure simulating 3000 m asl, FiO_2_ of 0.14.

Test M3: CPR at normal pressure simulating 5000 m asl, FiO_2_ of 0.11.

Prior to M2 and M3, each participant will have an acclimatisation period of 10 min to FiO_2_ corresponding to each of the altitudes.

To minimise the effect of fatigue between tests, each participant will have a 24 h rest period.

Stage 3: Participants assigned to the intervention group will undergo a standardised intermittent hypoxia conditioning programme (13 sessions, 45–75 min/session), including alternating cycles of hypoxia (FiO_2_ progressively reduced from 0.14 to 0.11) and normoxia, resulting in a total hypoxic exposure of approximately 728 min. The control group will undergo a sham protocol consisting of identical session structure and duration under normoxic conditions. Three to five weekly sessions will be held according to tolerance and the iAltitude^®^ protocol [42].

Stage 4: After the intervention, all the participants (*n* = 56) will perform three uninterrupted 10 min sessions of CPR: the first at 670 (P1), the second (P2) at 3000, and the third (P3) at 5000 m asl. To minimise the effect of fatigue between tests, each participant will have a 24 h rest period [42]. The criteria for completing the different CPR tests will be determined by reaching 10 min or by the physical and/or physiological limitations of the rescuer (physical exhaustion or pain, SatO_2_ < 75).

Throughout all stages, heart rate and peripheral oxygen saturation (SpO_2_) will be continuously monitored using an ear clip pulse oximeter. The training programme instructs the participant to breathe ambient air if the SpO2 level exceeds the limit specified in Table 1 (Lim SpO2). During hypoxia tests, as well as during conditioning, a nurse and/or a doctor with experience in emergency care will be present.

To minimise potential confounding due to independent practice, participants will be explicitly instructed not to perform any additional CPR training or practice outside the study protocol during the study period.

The pre-conditioning protocol for high-altitude exposure was designed based on experience and knowledge regarding the use of normobaric intermittent hypoxia (IHT) to enhance sporting performance. To this end, the following data were collected from each participant: age, sex, height, weight, body fat percentage, sport practised (if applicable), number of weekly training sessions, time spent training, and ability to perform CPR manoeuvres in accordance with the study design.

**Figure 2:**
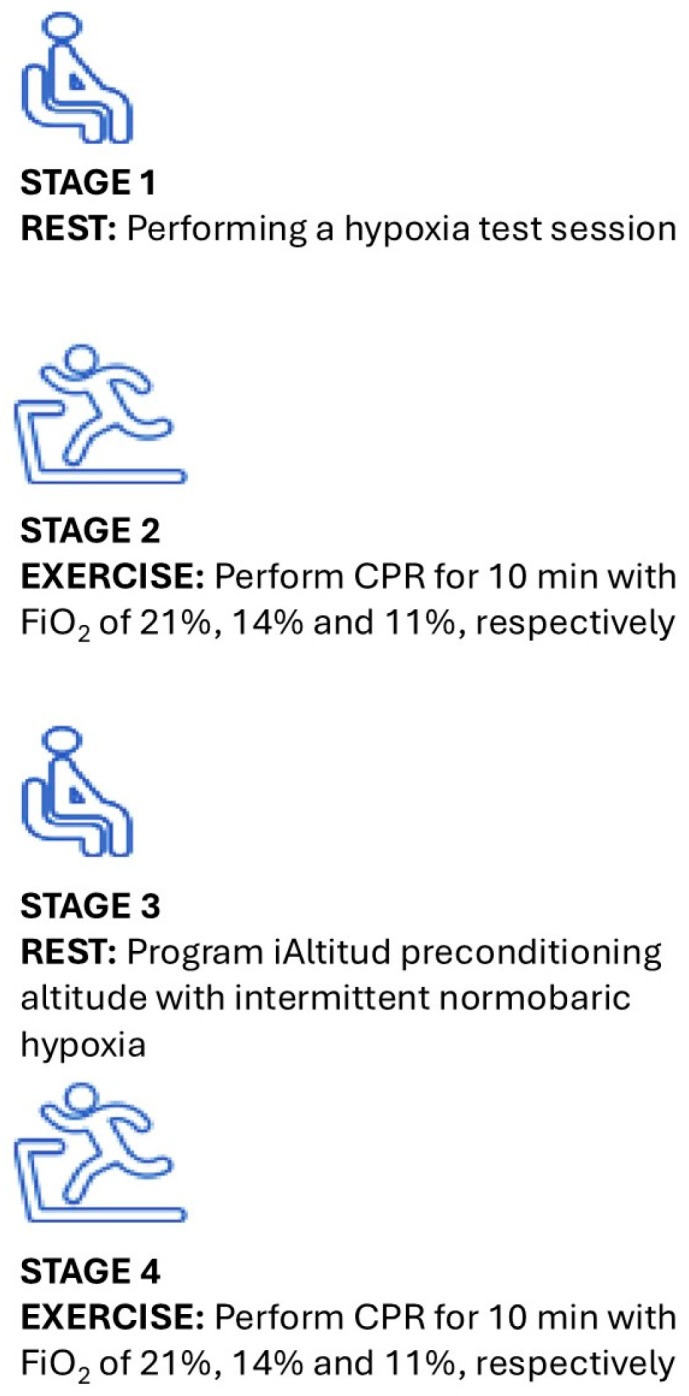
Pre-conditioning protocol. FiO_2_: fraction of inspired oxygen.

As indicated in the text and illustrated in Figure 2, the study protocol comprises four stages. The first stage involves performing a hypoxia tolerance test. The second stage involves carrying out CPR tests without hypoxia conditioning at different altitudes. The third stage consists of 15 sessions of IHT conditioning. Finally, the fourth stage involves performing CPR tests at the specified altitudes.

The Hypoxic Training Index (HTI) and the Hypoxic Recovery Index (HRI) are monitored in both phase 1 and phase 3. The HTI is a measure of the cumulative hypoxic stress experienced during a session and over successive sessions of the protocol. This index is calculated as an integral value of SPO2 readings below 90%. The HRI is used as an indicator of the individual’s recovery capacity following hypoxic stress during a session, and cumulatively across subsequent sessions of the protocol. It is calculated during periods when SPO2 readings are above 90%, and its value increases more rapidly the better an individual recovers from the hypoxic load to which the individual is exposed. The HTI is calculated as the area under the curve (AUC) of SpO_2_ values below 90%, expressed as %·min, representing the cumulative magnitude and duration of hypoxic stress. The HRI is calculated as the area above 90% SpO_2_ during recovery periods (also expressed as %·min), reflecting the extent and rate of reoxygenation.

In order to complete the designed IHT pre-conditioning protocol, participants will need to complete the 15 sessions detailed in Table 1. These sessions will be conducted under the supervision of a trained supervisor who will be responsible for ensuring that: (1) throughout all sessions, participants are monitored via pulse oximetry and follow the instructions provided by the iAltitude software (Hypoxiahome Trainer 4.1) at all times in order to ensure safety and achieve the expected results; (2) a minimum interval of at least 24 h is maintained between sessions, with no more than 72 h elapsing between them; (3) more than 2 h have elapsed between the participant’s last meal and the start of the session; and (4) the breathing pattern during the sessions remains natural. The breathing rate should not be increased, and the breaths should not be deep, so that the amount of oxygen inhaled corresponds to the level required for the session, based on the specified FiO_2_ percentage.

SPO2 Limit: This value indicates how many times the SPO2 has exceeded the safety limit set for the current session. The lower limit for blood oxygen saturation is set for each session in line with its objective. Whenever the limit is exceeded, the software alerts the user to remove their mask and recover in normoxia until they receive a new notification to put the mask back on.

### 2.5. Study Variables

Sociodemographic variables (age, sex and place of residence) will be measured in all participants.

BMI: The quotient of weight (kg) and height^2^ (m). BMI will be categorised according to the age and gender cut-off points defined by the World Health Organization in underweight (<18.5 kg/m^2^), normal weight (18.5–24.9 kg/m^2^) and overweight/obesity (≥25 kg/m^2^) [43].

Body fat percentage: A calibrated multifrequency signal bioimpedance meter with electrodes on the feet and a grip on both hands will be used for measurement. The participant will remain barefoot with the heels and soles of the feet located on the sensors of the equipment and the grip of the hands in the most appropriate grip position for their height. The bioimpedance analysis will be performed after a fast of at least 5 h, without having performed physical exercise or ingested alcohol in the previous 48 h and having previously emptied the urinary bladder—Seca-770 (SECA Gmbh & Co. Kg, Hamburg, Germany).

Muscle strength: Measured with a Takei TKK 5101 (Takei Scientific Instruments Co., Ltd., Tokyo, Japan) dynamometer, the muscular strength will be categorised as excellent, good, medium, bad or very bad. Muscular strength will be dichotomised, taking the cut-off point of the 25th percentile, by sex.

CPR quality: Upon performing the CPR manoeuvre, on the basis of previous studies [44,45], quality CC (Q-CC) will be evaluated via the following formula:

Q-CC (%) = [% CCcD + % CCcR + % CCchR]/3.(1)

In the equation, CCcD is the CC with correct depth (between 5 and 6 cm), CCcR is the CC with correct rhythm (100-/min) and CCchR is the CC with complete chest re-expansion.

Effective ventilation (EV) is evaluated as

EV (%) = (NEV × 100)/NTV(2)

where NEV is the number of effective ventilations and NTV is the total number of ventilations.

These parameters are based on the ERC-2021 [9] recommendations and are recorded by phantom software (Laerdal Resusci Anne CPR-D and SkillRepoter^®^; Medical Laerdal; Stavanger, Norway).

Adequate self-perception of CC and ventilations: Asking the participants if they think they have performed them correctly, with the possible answers being CC: YES/NO, V: YES/NO.

The results will be tabulated at different altitudes.

SO_2_m: This will be measured with near-infrared spectrophotometry in biceps brachii and erector spinae. The data will be categorised into four phases: baseline, phase close to fatigue, the fatigue phase and the recovery phase—Humon Hex® (Humon Inc., Cambridge, MA, United States).

VO_2_max: Using ergospirometry, this will be categorised as very bad, bad, medium, good and very good level of cardiorespiratory fitness. It will be measured with a treadmill ergometer (HP-Cosmos, mod. Pulsar 3P) (h/p/cosmos sports & medical GmbH, Nussdorf-Traunstein, Germany), Cardinal Health electrocardiograph mod. Ergoline ER800 (ergoline GmbH, Bitz, Germany) and an automatic breathing gas analysis system (mod. Oxycom Alpha-Jaeger) (Erich Jaeger GmbH, Höchberg, Germany).

Cardiorespiratory fitness will be dichotomised as higher and lower using the Cooper Institute cut-off points for 20–30-year-old adults (VO_2_max ≥38 mL/kg/min for women and ≥43 mL/kg/min for men) [46].

Blood determinations: Before the start and after the completion of each test (pre and post) Hgb, Hct, Mb, and lactate levels in venous blood will be determined with Epoc^®^ Blood Analysis System devices (Siemens Healthineers, Ottawa, ON, Canada).

RPE: At the end of each of the CPR tests, the participants will be asked to rate the perception of the effort made using the Borg scale. It consists of numbers from 6 to 20, where each number represents a level of perceived exertion. The number 6 on the scale is associated with very light effort, whereas the number 20 is associated with maximum effort. The intermediate numbers on the scale represent increasing levels of perceived exertion [47].

Anxiety levels: The State–Trait Anxiety Inventory (STAI) questionnaire will be used [48]. This questionnaire measures anxiety in healthy adults. It consists of two scales: SA (which reflects the subjective and transitory feelings of tension, apprehension and fear that can vary with time and fluctuate in intensity) and TA (which reflects a relatively stable anxious propensity that characterises individuals with a tendency to perceive situations as threatening). The questionnaire provides a numerical value for the TA and another for the SA. The total STAI is the sum of SA and TA. Each subscale is made up of a total of 20 items in a 4-point Likert response system according to intensity (0 = almost never/not at all; 1 = somewhat/sometimes; 2 = quite/often; 3 = a lot/almost always). The total score for each of the subscales ranges from 0 to 60 points. STAI-TA will be administered at the beginning of the investigation and STAI-SA at the end of pre-conditioning and post-conditioning CPR at 670 m asl and 5000 m asl.

### 2.6. Statistical Analysis

First, a descriptive analysis of the study variables will be carried out, along with the appropriate tabulation and graphical representation of the data. Qualitative variables will be expressed as proportions with 95% confidence intervals, and quantitative variables will be described using means and standard deviations or medians and interquartile ranges, as appropriate. Prior to the application of parametric tests, the assumption of normality will be assessed for all continuous variables using the Shapiro–Wilk test.

For comparisons involving paired quantitative data, Student’s *t*-test for paired samples will be used when the normality assumption is met. If this assumption is violated, the non-parametric Wilcoxon signed-rank test will be applied.

For between-group comparisons (intervention vs. control), independent-sample t-tests will be used for normally distributed variables, while the Mann–Whitney U test will be used for non-normally distributed variables.

To evaluate the effect of the intervention across time and altitude conditions, a mixed-model ANOVA will be used, including group (intervention vs. control) as a between-subject factor and time (pre vs. post) and altitude as within-subject factors.

For categorical variables, McNemar or chi-square tests will be applied as appropriate.

A multivariate linear regression analysis will be performed to adjust for potential confounding variables (age, sex, BMI, and physical fitness), including group allocation as an independent variable. Model assumptions (normality, homoscedasticity, and independence of residuals) will be checked.

Statistical significance will be set at *p* < 0.05. All statistical analyses will be performed using IBM^®^ SPSS^®^ Statistics 28 software.

## 3. Discussion

The results of this study will have the practical utility of demonstrating that CPR, even though it is a submaximal effort manoeuvre, is subject to being performed by anyone without motor disabilities regardless of the environment in which it is performed. Our study will also provide evidence as to whether this characteristic continues to hold true in a hostile environment such as medium and high altitudes.

Our study aims to demonstrate that improvement in physical performance and recovery capacity induced by intermittent hypoxia conditioning programmes increases the quality of CPR in prolonged cardiac arrests and in adverse conditions, such as at high altitudes.

In the same way, information will be given about how often the exchange of resuscitators is recommended to avoid errors in CPR manoeuvres as a result of rescuer fatigue under such adverse conditions.

The proposed study will contribute as a novelty to the estimation of the influence of high altitudes and conditioning on performing basic CPR manoeuvres.

If the hypothesis turns out to be true, recommendations about the practice of moderate-intensity physical exercise could be incorporated into the CPR guidelines as one of the important aspects in the training of rescuers to face CPR.

Limitations of this study include the fact that CPR will be performed not on a patient, but on a mannequin. However, a better performance under “real” conditions would have been more likely to occur since chest compressions are easier to perform on human beings. Similarly, simulating high altitude by reducing FiO_2_ in the laboratory eliminates other factors associated with high altitude, such as ambient temperature and reduced O_2_ pressure, the time of year of assessment, geographical conditions, the type and quality of food and the presence of unhealthy habits. On the other hand, the laboratory environment is very different from what usually surrounds sudden cardiac arrest in the community setting, and rescuers might vary their response when a sense of urgency appears.

A second limitation could be the effect that real-time audio-visual feedback during CPR manoeuvres has on the performance of the study participants, which may limit the generalisability of our findings to situations where this feedback procedure is not used.

Another limitation is the potential for bias when using an analytical pre–post comparison study, as well as the need for further validation of the generalisability of the results due to the small sample size.

It is worth mentioning the difficulty of conducting a trial with a large number of participants among the obstacles to achieving the planned objectives. As a consequence, the trial’s internal validity is threatened by the lack of representativeness of the sample. Another threat may be errors in the measurements; to minimise them, the variability of the interviewers and the nurses responsible for the measurements will be minimised through previous training [8,9].

## 4. Conclusions

The results of this study will have the practical utility of demonstrating that CPR can be performed by anyone without motor disabilities, regardless of the environment in which it is performed.

## Figures and Tables

**Table 1:** Description of normobaric hypoxia training sessions.

Session Number	FiO_2_	Lim SpO_2_	Total Cycle Time (min)	Hypoxia Cycle Duration (min) [Number of Hypoxia Cycles]	Normoxia Cycle Duration (min) [Number of Normoxia Cycles]	Total Hypoxia Time (min)	Total Normoxia Time (min)
1	14	85	56	4 [7]	4 [7]	28	28
2	14	85	64	5 [8]	3 [8]	40	24
3	14	85	63	6 [7]	3 [7]	42	21
4	13	82	64	5 [8]	3 [8]	40	24
5	13	82	70	7 [7]	3 [7]	49	21
6	13	82	63	7 [7]	2 [7]	49	14
7	12	80	64	6 [8]	2 [8]	48	16
8	12	80	70	7 [7]	3 [7]	49	21
9	12	80	60	8 [6]	2 [6]	48	12
10	13	82	72	16 [4]	2 [4]	64	8
11	11	78	63	7 [7]	2 [7]	49	14
12	11	78	60	8 [6]	2 [6]	48	12
13	11	78	60	9 [6]	1 [6]	54	6

FiO_2_: fraction of inspired oxygen; Lim SpO_2_: arterial blood oxygen saturation limit.

## Data Availability

The original contributions presented in this study are included in the article. Further inquiries can be directed to the corresponding author.

## Abbreviations

The following abbreviations are used in this manuscript:

AHA	American Heart Association
asl	Above sea level
BMI	Body mass index
CA	Cardiac arrest
CC	Chest compression
CPK	Creatine phosphokinase
CPR	Cardiopulmonary resuscitation
DBP	Diastolic blood pressure
ERC	European Resuscitation Council
FiO_2_	Oxygen fraction inspirated
Hct	Haematocrit
Hgb	Haemoglobin
HR	Heart rate
Mb	Myoglobin
METs	Metabolic equivalents
NEV	Number of effective ventilations
NTV	Total number of ventilations
OHCA	Out-of-hospital cardiac arrest
RPE	Rating of perceived exertion
SaO_2_	Arterial oxygen saturation
SBP	Systolic blood pressure
SmO_2_	Muscular oxygen saturation
